# Biographical Sketches

**Published:** 1902-06

**Authors:** 


### BIOGRAPHICAL SKETCHES.

Our readers have doubtless been interested in the sketches of prominent men in dentistry presented from time to time by Dr. Charles McManus, of Hartford, and the half-tone reproductions that have accompanied these as frontispiece illustrations. The value of Dr. McManus's work can hardly be over-estimated in that he is giving to the present generation of dentists a vivid conception of the life and appearance of the men who made dentistry in this country and, it is hoped, before he concludes, of the world

In the present issue is presented a very striking picture of Edward Hudson. We are indebted for this to Dr. E. T. Darby, who, after much difficulty and a good deal of diplomacy, succeeded in securing the consent of relatives to have a photograph taken of the portrait by Sully.

The character of the man as portrayed by Dr. McManus is strikingly illustrated in this picture, a marked individuality of character accompanied by all the characteristics of refinement peculiar to the cultured men of the period.

Dr. Hudson is almost an unknown figure in dental history, for the reason that he was not a book-maker, and dental journals were not in existence during his lifetime of work. His reputation has been, therefore, almost purely local, and dependent largely on tradition, but this has been of great value as it demonstrates a well-attested fact, that at least one remarkably skilled operator existed in the early years of the nineteenth century and that all the good workers in gold have not been concentrated in the last decade of the same century or the first of the twentieth.

Those who were contemporary with Dr. Elisha Townsend, an operator without a peer in his day, know the value he set upon Dr. Hudson's work. While he may not have known Hudson personally, he had ample opportunities of examining his work, for these splendid efforts were quite familiar to dentists of Philadelphia fifty years after insertion.

From an old Philadelphia Directory of 1814, in possession of the writer, Dr. Hudson's address is given as 133 Walnut Street (old style), at that time the best residential quarter in that city, but now given up exclusively to business. In this year there were only thirteen dentists in that city, but among that small number were several names, besides that of Hudson, that have come down to us with honor, such as Gardette, Gilliams, Koecker, and Van Pelt. When this period is compared with the present time the progress in dentistry becomes more apparent. The present number of dentists in Philadelphia will not fall far short of seven hundred. Polk's Dental Directory for 1900 gives it six hundred and fifty-four. Add to this number four dental colleges, with thirteen hundred students, and the advance made in a hundred years becomes almost startling. If the next century, our present twentieth, enlarges its work in the same proportion, it is possible that it will mark a revolution in conditions and methods.

The work of Dr. McManus in the INTERNATIONAL, and of Dr. Thorpe in the *Dental Review,* gives encouraging evidence that a few are interested in dental history and are determined to rescue it from unmerited oblivion. It should, therefore, be possible in the near future to have a History of Dentistry worthy the name.

The men of activity in the past century are rapidly disappearing from the world's life. While personal knowledge is not absolutely essential as an aid to the writers of such a history, it gives a vivid charm to otherwise dry compilations that nothing else can furnish. This history has been talked about since the Columbian meeting, when a committee failed to make a proper report, and it is still in the hands of a more competent committee of the National Dental Association, of which Dr. McManus is chairman. It will necessarily be a work of years, but if the labor be divided up into several periods, with interested collaborators at the head of each, the result would be more satisfactory. This history is so badly needed that the writer would be gratified if he could anticipate the publication of the work in the near future.

